# Adolescent acne: association to sex, puberty, testosterone and dihydrotestosterone

**DOI:** 10.1530/EC-25-0009

**Published:** 2025-04-02

**Authors:** Nanna E Jakobsen, Jørgen Holm Petersen, Lise Aksglaede, Casper P Hagen, Alexander S Busch, Trine Holm Johannsen, Hanne Frederiksen, Anders Juul, Stine A Holmboe

**Affiliations:** ^1^ Department of Growth and Reproduction, Copenhagen University Hospital – Rigshospitalet, Copenhagen, Denmark; ^2^ International Center for Research and Research Training in Endocrine Disruption of Male Reproduction and Child Health (EDMaRC), Copenhagen University Hospital – Rigshospitalet, Copenhagen, Denmark; ^3^ Department of Biostatistics, University of Copenhagen, Copenhagen, Denmark; ^4^ Department of Clinical Medicine, University of Copenhagen, Copenhagen, Denmark

**Keywords:** acne, puberty, dihydrotestosterone (DHT), testosterone

## Abstract

The manifestation of acne in adolescents coincides with the emergence of other androgen-dependent characteristics of puberty such as sweat odor and pubic hair. Yet, little is known about the associations with circulating levels of androgens. Thus, the objective was to study the prevalence of acne in healthy children and adolescents according to sex, age, pubertal stage and concentrations of testosterone and dihydrotestosterone (DHT) quantified by LC-MS/MS. This is a secondary analysis of a larger study on puberty. Data included a combined cross-sectional and longitudinal population-based cohort study, including 1,609 participants from public schools (aged 5.6–23.4) who were assessed for acne. Of these, 222 participants were examined every 6 months for 8 years. In a nested cohort of the cross-sectional population (*n* = 1,009), concentrations of testosterone and DHT were measured. To determine age at onset of acne, probit analyses were performed, integrating left-, right- and interval-censored data to estimate the mean age at which acne was recorded. In boys, acne occurred at a mean age of 15.0 years (95% CI: 14.7–15.3) based on probit analyses, whereas such analyses could not be performed in girls due to insufficient numbers of girls with acne. Acne was observed in boys in Tanner stages G4 (44%) and G5 (83%) and was less frequent in girls in stages B4 (15%) and B5 (12%). DHT was significantly higher in boys and girls with current acne compared to adolescents without. In conclusion, the prevalence of acne was 85% in late pubertal boys and 15% in late pubertal girls. DHT concentrations were higher in adolescents of both sexes presenting with acne than in those without.

## Introduction

Puberty is a complex and dynamic process resulting in children transitioning into adults with reproductive capabilities. Puberty is initiated by an increase in pulsatile gonadotropin-releasing hormone (GnRH) release from the hypothalamus, which stimulates the pituitary release of luteinizing hormone (LH) and follicle-stimulating hormone (FSH). LH and FSH subsequently stimulate the production of gonadal hormones including estradiol and testosterone, which are responsible for the development of secondary sex characteristics (1). Testosterone production by the theca cells in girls and by Leydig cells in boys stimulate androgen-responsive tissues through activation of the androgen receptor. Importantly, testosterone is converted by 5α-reductase to dihydrotestosterone (DHT), which is expressed in numerous tissues including the sebaceous glands of the skin (2). Testosterone and DHT constitute the main androgens, with DHT being the most potent with a significantly higher affinity for the androgen receptor than testosterone (3). In adult men, the serum concentration of DHT is about one-tenth of the total serum testosterone concentration, whereas in adult women, serum concentrations are generally lower and the differences in concentrations are less pronounced (4).

The onset of puberty is marked by some distinctive, well-defined clinical characteristics such as estrogen-dependent breast development in girls, FSH-dependent testicular enlargement in boys, androgen-dependent enlargement of penis in boys and pubic hair development in both sexes. These characteristics are, along with increasing growth velocity, some of the most visible changes during puberty. However, other well-known androgen-dependent pubertal milestones such as sweat odor, acne vulgaris and mood changes have received less attention (5, 6).

Acne vulgaris, or acne, is estimated to affect 9.4% of the entire population, although substantial differences in prevalence across age groups exist (7, 8). The onset of acne is typically correlated with puberty, with the highest prevalence seen in adolescence (9). Besides symptomatic discomfort, acne is associated with emotional and psychosocial distress and has been associated with an increased risk of anxiety, depression and suicidal ideation (10). Before puberty, there is minimal difference in circulating concentrations of testosterone between boys and girls (4). At puberty, the pulsatile increase in both amplitude and frequency of GnRH and LH causes an increase in the production of testosterone, with boys having higher concentrations compared to girls. In both sexes, testosterone concentrations increase significantly with each pubertal stage (11). The clinical usefulness of quantification of circulating DHT concentrations remains unclear, except in the case of patients suspected of 5α-reductase deficiency (12). Furthermore, the clinical use of 5α-reductase inhibitors such as finasteride, which reduces DHT, has been successfully applied in management of acne in adult women (13).

Several studies have shown that the prevalence of acne increases with pubertal maturation (9, 14, 15). However, controversy exists regarding the association between circulating levels of androgens and pubertal acne (15, 16, 17). Differences in findings are likely due to factors such as differences in the clinical evaluation of acne, the previous use of insensitive methods for quantification of androgens and the differences in ethnicity of the included populations. To our knowledge, the potential association between circulating concentrations of testosterone and DHT measured by ultrasensitive liquid chromatography–tandem mass spectrometry (LC-MS/MS) and risk of acne in healthy adolescent boys and girls has not yet been investigated.

Therefore, we aimed i) to describe the prevalence and timing of acne during puberty in healthy Danish children and adolescents and ii) to investigate the association between presence of acne and serum concentrations of testosterone and DHT analyzed by LC-MS/MS.

## Material and methods

### Study population

The present study on acne is a secondary analysis of a larger study on puberty. All participants were recruited as part of the Copenhagen Puberty Study, a combined cross-sectional and longitudinal population-based cohort study conducted at primary schools in Copenhagen in the period from 2006 to 2014 (ClinicalTrials.gov ID: NCT01411527) (18, 19). In total, 1,609 participants with information on acne available were included in the present study (58% girls, 42% boys) ranging from 5.6 to 23.4 of age. Of these, 222 children and adolescents were part of a longitudinal study and were examined every 6 months for 8 years. In a subset of the cross-sectional population (*n* = 1,009 participants, 57% girls, 43% boys), serum concentrations of testosterone and DHT were analyzed. The study was approved by the ethical committee of The Capital Region of Denmark (no. KF01282214) and the Danish Data Protection Agency (no. 2015-41-4494). All participants and their parents gave informed consent before their participation in the study.

### Clinical examination

Pubertal stages were assessed by trained physicians according to the methods of Marshall and Tanner (20, 21). According to these criteria, breast stage (B1-B5) and pubic hair stage (PH1-PH5) were evaluated in girls, and genital stage (G1-G5) and pubic hair stage (PH1-PH5) were evaluated in boys. The presence of current acne was clinically evaluated by trained physicians and recorded as present or not (yes/no). The physicians assessing acne were aligned regarding the threshold of acne, and the participants had to have more than a few red spots/pimples (<2–3) before being recorded as having acne. Furthermore, girls were asked whether they currently used any types of oral contraceptives (OCs) (yes/no) at the time of examination.

Standing height was measured to the nearest 0.1 cm using a wall-mounted stadiometer, and the participants were weighed on a digital electronic scale (Seca delta, Germany) with a precision of 0.1 kg. Body mass index (BMI) was calculated as weight in kilograms divided by height in meters squared. Blood sampling was performed in the morning and serum samples were stored at −20°C until further analyses. Written informed consent was obtained from all participants.

### Quantification of testosterone and DHT

Serum concentrations of testosterone and DHT were determined by a newly developed method for simultaneous quantification of 16 steroid hormones by online-TurboFlow-LC-MS/MS with prior isotope-dilution as recently described in detail (4). The limits of detection (LODs) were 0.031 nmol/L for testosterone and 0.118 nmol/L for DHT. Samples for this sub-study were analyzed in 20 batches over a period of 3 months. Besides cohort samples, each batch included calibrations samples, blanks (water) and three times three control samples of stripped human serum spiked with a native standard mixture in two different levels (Q1 and Q2) and human serum which was a pool of leftover serum from patients. The inter-day variation given as the relative standard deviations (RSDs) were ≤5.5% for testosterone, while RSDs for DHT were 10% (Q1), 5.5% (Q2) and 19% (serum pool). External quality control (UK NEQAS) for testosterone revealed excellent performance when compared with other laboratories using LC-MS/MS technology. The analysis of DHT has not yet been committed to an external control program.

### Statistical methods

In the cross-sectional study, standard deviation (SD) scores for height, weight, BMI and serum concentrations of testosterone and DHT were calculated by generalized additive models for location, scale and shape (GAMLSS) methodology, allowing comparison across sex and age. Reference charts were developed using GAMLSS statistics as described in detail (4). Differences in age, anthropometric measures and sex steroids stratified according to the presence of acne were tested using the Mann–Whitney U test. In sub-analyses, the differences were tested in a restricted population only including children in the physiologically relevant age range where acne was present (girls ≥9 years of age and boys ≥12 years of age).

To determine the age at the onset of acne in the combined cross-sectional and longitudinal study, probit analyses were performed, integrating left-, right- and interval-censored data to estimate the mean age at which acne was recorded (22). Longitudinal measurements were used as interval-censored data, where first occurrence of acne was included. Thus, in individuals with examinations both before and after occurrence of acne, the interval contained the exact age of acne. Cases without experience of acne were treated as right-censored data, where only the lower bound for the true age of onset was recorded. Cases with acne at the first examination were treated as left-censored data, where only the upper bound for the true age of onset was recorded.

Finally, to investigate the impact of age, pubertal stage (breast stage/genital stage) and BMI on the presence of current acne in boys and girls separately, a multiple logistic regression analysis was used by applying a generalized estimating equation model, the latter allowing more than one measurement per individual to take into account that some children were examined longitudinally whereas others were not (23). In sub-analyses, to investigate the potential impact of use of OC in girls, the difference in distribution of OC users according to acne status was tested using chi-square test, and differences in SD scores of testosterone and DHT stratified according to self-reported OC use were tested using the Mann–Whitney U test. DHT values below LOD (1.6%) were treated as the LOD value divided by 2. The testosterone concentration was >LOD in all participants.

All statistical analyses were generated using the IBM SPSS Statistics 28 and RStudio-2023.03.0-386. *P*-values<0.05 were considered statistically significant.

## Results

**Figure 1:**
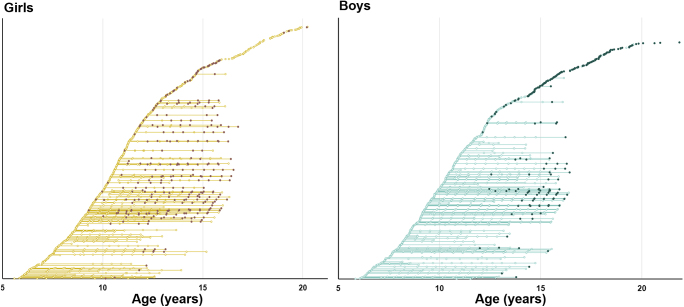
Combined longitudinal and cross-sectional overview (*n* = 1,609) of age at examination stratified according to current acne (929 girls and 680 boys). Filled dots indicating presence of acne.

**Figure 2:**
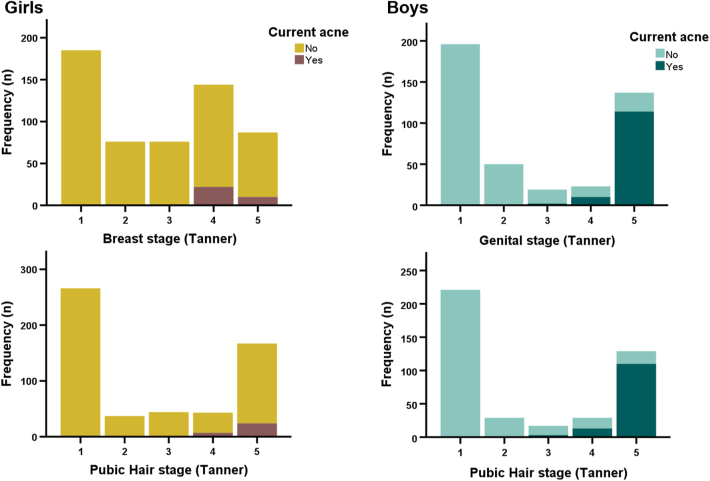
Cross-sectional study population (*n* = 1,009). Distribution of participants according to Tanner stages and stratified according to current acne status (571 girls and 438 boys).

In the combined longitudinal and cross-sectional study population based on 1,609 individuals, acne was present in more boys (26.0%) than girls (11.8%) at the time of examination (Fig. 1). In boys, the mean age at presentation of acne was 15.0 years (95% CI: 14.7–15.3 years). In girls, the mean age could not be determined by probit analysis due to their generally lower prevalence of acne. However, cross-sectionally, girls with acne were younger than boys with acne, 14.8 vs 16.6 years, respectively (Table 1). Among the girls with self-reported information on OC use (*n* = 551), 15% of girls with acne (*n* = 5/33) also reported use of OC compared to 9% (*n* = 49/518) of girls without acne (*P*-value = 0.36). In both sexes (*n* = 1,009), acne was present exclusively in late pubertal stages (Fig. 2). Thus, acne was present in 83% of boys in Tanner stage G5 and 85% in stage PH5, respectively. In girls, acne was present in 15% of girls in stage B4, 12% in stage B5, 16% in stage PH4 and 14% in stage PH5, respectively. Height and weight SD-scores were significantly higher in boys and girls with current acne compared to those without (Table 1). Furthermore, girls with acne had higher BMI SDS compared with girls without (0.37 vs 0.17, *P* = 0.05), whereas no difference in BMI SDS was seen in boys (0.28 vs 0.25, *P* = 0.97). Comparable findings were observed in subanalyses including only girls ≥9 years and boys ≥12 years (data not shown).

**Table 1:** Cross-sectional study population. Descriptive statistics (median (IQR) and *n* (%)) according to sex and current acne status.

	Girls (*n* = 571)			Boys (*n* = 438)		
	Current acne		*P*-value*	Current acne		*P*-value*
	Yes	No		Yes	No	
*n* (%)	35 (6.1)	536 (93.9)		135 (30.8)	303 (69.2)	
Age (years)	14.8 (12.9; 15.5)	11.2 (9.3; 14.1)	<0.01	16.6 (15.1; 18.0)	10.4 (8.8; 12.3)	<0.01
Height (SD score)	0.35 (−0.52; 0.72)	−0.13 (−0.72; 0.57)	0.05	0.52 (−0.29; 1.09)	−0.19 (−0.82; 0.48)	<0.01
Weight (SD score)	0.50 (−0.09; 0.94)	0.05 (−0.58; 0.69)	0.01	0.41 (−0.19; 0.94)	0.04 (−0.70; 0.70)	<0.01
BMI (SD score)	0.37 (0.01; 0.96)	0.17 (−0.51; 0.78)	0.05	0.28 (−0.36; 0.72)	0.25 (−0.52; 0.87)	0.97
Testosterone (SD score)	0.13 (−0.71; 1.06)	0.09 (−0.64; 0.78)	0.43	0.44 (−0.10; 1.14)	−0.15 (−0.81; 0.67)	<0.01
DHT (SD score)	0.29 (−0.44; 1.73)	−0.04 (−0.68; 0.57)	0.02	0.29 (−0.38; 0.93)	−0.13 (-0.70; 0.50)	<0.01
OC use	5 (15.2)	49 (9.5)	0.36			

* *P*-values based on Mann–Whitney U test or chi-squared test.

Abbreviations: IQR, interquartile range; BMI, body mass index; SD, standard deviation; DHT, dihydrotestosterone; OC, oral contraceptive.

Based on the combined longitudinal and cross-sectional study population (*n* = 1,609), age was strongly associated with odds of having acne, with a more pronounced effect in boys; OR (95% CI) = 2.9 (2.4–3.4) than in girls; 1.5 (1.4–1.6). Similarly, higher odds of having acne were seen with higher pubertal stage both in boys; OR = 5.6 (4.6–6.9) and in girls; 2.4 (2.0–3.0). Furthermore, in age-adjusted analyses, a higher BMI was significantly associated with the presence of acne in boys; OR = 1.4 (1.0–1.8), *P* = 0.02, but not in girls; OR = 1.1 (0.8–1.4), *P* = 0.45. However, when adjusting for pubertal stage instead of age, the association for BMI disappeared.

**Figure 3:**
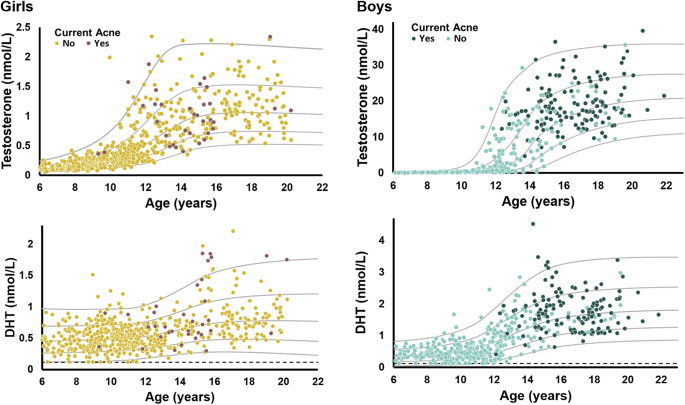
Cross-sectional study population (*n* = 1,009). Concentrations of testosterone and dihydrotestosterone (DHT) in 571 girls (yellow) and 438 boys (green) stratified according to current acne status (no/yes).

**Figure 4:**
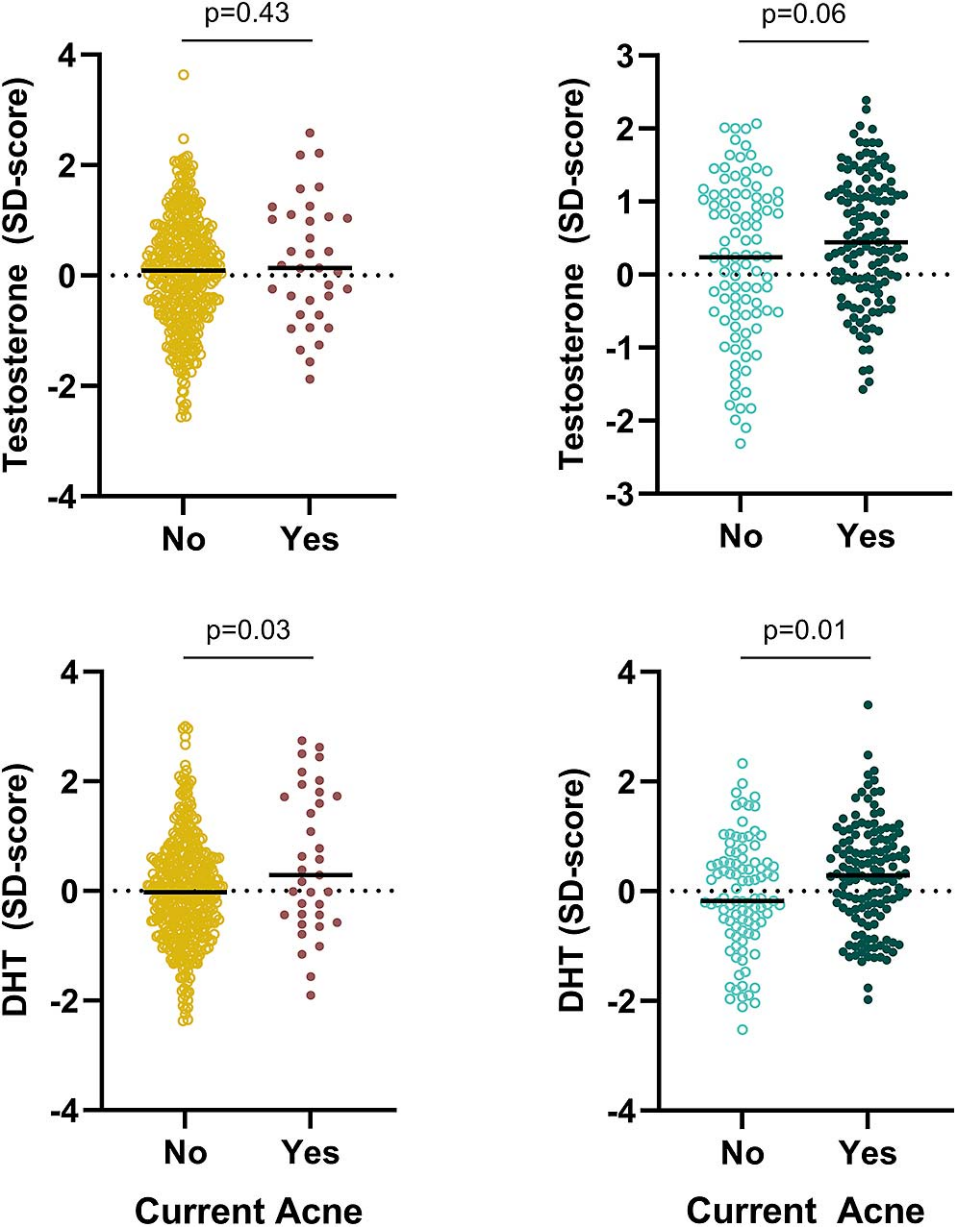
Cross-sectional study population (*n* = 681). Subanalyses of girls above age 9 (*n* = 452) and boys above age 12 (*n* = 229). SD scores for serum concentrations of testosterone and dihydrotestosterone (DHT) according to current acne status (no/yes), respectively. Figure text: differences in the median value tested using the Mann–Whitney U test. One outlier excluded on graph with testosterone for girls (testosterone SD-score = −5.7 in the group with no current acne).

In the cross-sectional study population including all age groups, boys and girls with acne had higher SD scores of DHT than adolescents without acne (girls: 0.29 vs −0.04 SDS, *P* = 0.02, boys: 0.29 vs −0.13 SDS, *P* < 0.01) (Table 1, Fig. 3). Furthermore, boys with acne had significantly higher testosterone SD scores than boys without (0.44 vs −0.15, *P* < 0.01). In restricted analyses, only including girls ≥ 9 years of age and boys ≥12 years of age, a similar tendency of higher DHT SDS in both sexes was seen in adolescents with acne compared with adolescents without (girls: 0.29 vs −0.02 SDS, *P* = 0.03, boys: 0.29 vs −0.18 SDS, *P* = 0.01) (Fig. 4). In addition, higher testosterone SDS in boys was seen although insignificant (0.44 vs 0.24 SDS, *P* = 0.06). In both sexes, there was a pronounced interindividual variation in SD scores for testosterone and DHT irrespective of acne status (Fig. 4). Finally, no statistical difference in the median SD scores of testosterone and DHT stratified according to the use of OC was seen (*P* = 0.15 and *P* = 0.25, respectively) (data not shown).

## Discussion

In this large clinical study of healthy children and adolescents, acne was present in 85% of late pubertal boys and in 15% of late pubertal girls; however, girls presented with acne at a younger age compared to boys. In both sexes, adolescents with the presence of acne had significantly higher concentrations of DHT than those without.

It has long been recognized that androgens can stimulate the activity of the sebaceous gland and thereby lead to acne (24, 25). A study reported that the androgenic effect of DHT is more potent than that of testosterone *in vitro*; however, the difference between these remains unclear (26). In addition, it has been reported that skin with acne converts testosterone to DHT two to twenty times more than skin without acne from a corresponding area (27). This may suggest an increased 5α-reductase activity in subjects who develop acne. In accordance, a small study reports a tendency of increased 5α-reductase activity in sebaceous glands in women with acne compared with women without, although insignificant. Furthermore, men had higher enzyme activity than women; however, there was no difference between men with and without acne (28). In the present study, we observed that serum testosterone levels were higher in boys with acne compared to those without, although only borderline significant (*P* = 0.06) in age-restricted analysis (Fig. 4). Furthermore, in both sexes, significantly higher DHT concentrations were observed in adolescents with acne compared to adolescents without. Thus, higher DHT concentrations could potentially be explained by an increased 5α-reductase activity causing the development of acne.

One of the most common causes of androgen excess in adult women is polycystic ovary syndrome (PCOS). The presence of acne is associated with PCOS due to biochemical hyperandrogenism (29). Furthermore, studies have indicated that the use of anabolic androgenic steroids may be involved in the development of inflammatory acne (30, 31). A recent study report that 58% of people using anabolic androgenic steroids suffer from acne (32). However, limited knowledge exists on acne development in relation to physiological concentrations of testosterone and DHT in healthy children and adolescents. A limited number of studies have investigated the potential differences in androgen levels between individuals with and without acne. Some studies have indicated that women with acne have higher concentrations of testosterone and DHT compared with women without (17, 28), but not all studies observe this (15, 16). Common for these studies is that androgens were analyzed using immunoassay methods, which are less specific, especially in the lower range, compared to mass spectrometry. Thus, it is likely that differences in the laboratory methods could explain some of the differences in findings.

A study reported that the concentration of estradiol is decreased in women with acne compared to controls (33). The role of estrogens in the pathophysiology of acne is not fully elucidated (34). However, it is well-known that estrogens suppress sebum production and decrease acne lesions by increasing sex hormone-binding globulin and thereby decrease the amount of circulating free testosterone (33, 34). Thus, higher estrogen levels relative to testosterone levels could possibly explain the sex difference in acne prevalence in the present study.

In the present study, girls tended to develop acne 1 to 2 years before boys, which is in accordance with other studies (14, 35). Earlier ages at presentation of acne in girls than in boys seem to be related to the earlier onset of puberty in girls. However, in the older age groups, clinical acne tends to be more commonly observed and more severe in boys than girls. This phenomenon has been speculated to be due to a higher sebum excretion in males than in females in adolescents (35). Furthermore, it could be speculated that the observed difference in the acne prevalence between boys and girls could be explained by older girls being more likely to use OCs, which are known to reduce the androgen levels (36), and thus could affect the likelihood of having acne. However, in the present study, no difference in prevalence of acne according to OC use or SD scores of testosterone and DHT were observed between OC users and non-users.

Height and weight SD scores were significantly higher in boys and girls with current acne compared to those without. Furthermore, in line with previous studies, the presence of acne was associated with higher BMI (37, 38). However, in the present study, the link between acne and BMI disappeared after adjusting for pubertal stage, suggesting that acne is related to pubertal stage rather than directly to BMI.

Premature adrenarche, defined as premature onset of adrenal androgen production, is often followed by phenotypic characteristics such as pubarche and axillary hair but also sweat odor and acne. Furthermore, in a case-control study, girls with premature adrenarche were more likely to have an unfavorable body composition, including higher BMI, compared to age-matched controls, highlighting the interplay between androgens and body composition (39). However, in the present study, we did not report adrenal androgen production in relation to the presence or absence of acne.

There are several strengths in the present study: i) sex steroid measurements were conducted by highly sensitive, state-of-the-art LC-MS/MS methodology; ii) the pubertal examinations were performed by medical doctors; iii) three trained physicians evaluated all subjects, thereby limiting interobserver variation; iv) only Caucasian participants were included, thereby excluding confounding by ethnic differences; and v) only healthy children recruited from public schools were included, thereby excluding selection bias from subjects referred to healthcare evaluation.

One limitation in the present study was the recording of acne, which was registered as being present or not, while no information on its severity according to a grading system was used. The prevalence of acne is difficult to compare between studies since no classification system has been accepted universally (40). Methods to study the severity of acne include everything from simple grading based on clinical examination and lesion counting to those requiring more complicated instruments such as video microscopy and fluorescent photography (41). The difficulty in assessing acne is related to the fact that acne is considered a highly pleomorphic disorder, thereby making it hard to define the severity (42). Besides the inclusion of data on OC use, another limitation of the study is the lack of information on medication, especially those known to affect sex hormones and/or medication used for acne treatment, which could potentially impact both the observed prevalence of acne and androgen levels in the cohort.

In summary, we report that pubertal acne is related to age, anthropometric measures, stage of pubertal development and, importantly, to a higher serum concentration of DHT.

## Declaration of interest

The authors declare that there is no conflict of interest that could be perceived as prejudicing the impartiality of the work reported.

## Funding

The study is funded by COPUS II fundings (Independent Research Fund Denmark; Region Hovedstaden, Kirsten and Freddy Johansen’s fond) and ASB is funded by the Deutsche Forschungsgemeinschaft (DFG, German Research Foundation) – 464240267.
